# 114 Social Participation Trajectories: Results from the LIBRE Journey Study

**DOI:** 10.1093/jbcr/irad045.087

**Published:** 2023-05-15

**Authors:** Jeffrey Schneider, Lauren J Shepler, Lewis Kazis, Mary D Slavin, Colleen Ryan, Pengsheng Ni, Amy Acton, Brian Kelter, Edward Santos

**Affiliations:** Spaulding Rehabilitation Hospital/Harvard Medical School/Rehabilitation Outcomes Center at Spaulding, Boston, Massachusetts; Spaulding Rehabilitation Hospital, Harvard Medical School, Boston, Massachusetts; Boston University School of Public Health, Rehabilitation Outcomes Center at Spaulding, Boston, Massachusetts; Department of Health Law, Policy and Management, Boston University School of Public Health, Boston, Massachusetts; Shriners Children's-Boston, Massachusetts General Hospital, Harvard Medical School, Boston, Massachusetts; Boston University School of Public Health, Boston, Massachusetts; Phoenix Society for Burn Survivors, Grand Rapids, Michigan; Massachusetts's General Hospital, Boston, Massachusetts; Spaulding Rehabilitation Hospital - Boston Harvard Burn Injury Model System, Boston, Massachusetts; Spaulding Rehabilitation Hospital/Harvard Medical School/Rehabilitation Outcomes Center at Spaulding, Boston, Massachusetts; Spaulding Rehabilitation Hospital, Harvard Medical School, Boston, Massachusetts; Boston University School of Public Health, Rehabilitation Outcomes Center at Spaulding, Boston, Massachusetts; Department of Health Law, Policy and Management, Boston University School of Public Health, Boston, Massachusetts; Shriners Children's-Boston, Massachusetts General Hospital, Harvard Medical School, Boston, Massachusetts; Boston University School of Public Health, Boston, Massachusetts; Phoenix Society for Burn Survivors, Grand Rapids, Michigan; Massachusetts's General Hospital, Boston, Massachusetts; Spaulding Rehabilitation Hospital - Boston Harvard Burn Injury Model System, Boston, Massachusetts; Spaulding Rehabilitation Hospital/Harvard Medical School/Rehabilitation Outcomes Center at Spaulding, Boston, Massachusetts; Spaulding Rehabilitation Hospital, Harvard Medical School, Boston, Massachusetts; Boston University School of Public Health, Rehabilitation Outcomes Center at Spaulding, Boston, Massachusetts; Department of Health Law, Policy and Management, Boston University School of Public Health, Boston, Massachusetts; Shriners Children's-Boston, Massachusetts General Hospital, Harvard Medical School, Boston, Massachusetts; Boston University School of Public Health, Boston, Massachusetts; Phoenix Society for Burn Survivors, Grand Rapids, Michigan; Massachusetts's General Hospital, Boston, Massachusetts; Spaulding Rehabilitation Hospital - Boston Harvard Burn Injury Model System, Boston, Massachusetts; Spaulding Rehabilitation Hospital/Harvard Medical School/Rehabilitation Outcomes Center at Spaulding, Boston, Massachusetts; Spaulding Rehabilitation Hospital, Harvard Medical School, Boston, Massachusetts; Boston University School of Public Health, Rehabilitation Outcomes Center at Spaulding, Boston, Massachusetts; Department of Health Law, Policy and Management, Boston University School of Public Health, Boston, Massachusetts; Shriners Children's-Boston, Massachusetts General Hospital, Harvard Medical School, Boston, Massachusetts; Boston University School of Public Health, Boston, Massachusetts; Phoenix Society for Burn Survivors, Grand Rapids, Michigan; Massachusetts's General Hospital, Boston, Massachusetts; Spaulding Rehabilitation Hospital - Boston Harvard Burn Injury Model System, Boston, Massachusetts; Spaulding Rehabilitation Hospital/Harvard Medical School/Rehabilitation Outcomes Center at Spaulding, Boston, Massachusetts; Spaulding Rehabilitation Hospital, Harvard Medical School, Boston, Massachusetts; Boston University School of Public Health, Rehabilitation Outcomes Center at Spaulding, Boston, Massachusetts; Department of Health Law, Policy and Management, Boston University School of Public Health, Boston, Massachusetts; Shriners Children's-Boston, Massachusetts General Hospital, Harvard Medical School, Boston, Massachusetts; Boston University School of Public Health, Boston, Massachusetts; Phoenix Society for Burn Survivors, Grand Rapids, Michigan; Massachusetts's General Hospital, Boston, Massachusetts; Spaulding Rehabilitation Hospital - Boston Harvard Burn Injury Model System, Boston, Massachusetts; Spaulding Rehabilitation Hospital/Harvard Medical School/Rehabilitation Outcomes Center at Spaulding, Boston, Massachusetts; Spaulding Rehabilitation Hospital, Harvard Medical School, Boston, Massachusetts; Boston University School of Public Health, Rehabilitation Outcomes Center at Spaulding, Boston, Massachusetts; Department of Health Law, Policy and Management, Boston University School of Public Health, Boston, Massachusetts; Shriners Children's-Boston, Massachusetts General Hospital, Harvard Medical School, Boston, Massachusetts; Boston University School of Public Health, Boston, Massachusetts; Phoenix Society for Burn Survivors, Grand Rapids, Michigan; Massachusetts's General Hospital, Boston, Massachusetts; Spaulding Rehabilitation Hospital - Boston Harvard Burn Injury Model System, Boston, Massachusetts; Spaulding Rehabilitation Hospital/Harvard Medical School/Rehabilitation Outcomes Center at Spaulding, Boston, Massachusetts; Spaulding Rehabilitation Hospital, Harvard Medical School, Boston, Massachusetts; Boston University School of Public Health, Rehabilitation Outcomes Center at Spaulding, Boston, Massachusetts; Department of Health Law, Policy and Management, Boston University School of Public Health, Boston, Massachusetts; Shriners Children's-Boston, Massachusetts General Hospital, Harvard Medical School, Boston, Massachusetts; Boston University School of Public Health, Boston, Massachusetts; Phoenix Society for Burn Survivors, Grand Rapids, Michigan; Massachusetts's General Hospital, Boston, Massachusetts; Spaulding Rehabilitation Hospital - Boston Harvard Burn Injury Model System, Boston, Massachusetts; Spaulding Rehabilitation Hospital/Harvard Medical School/Rehabilitation Outcomes Center at Spaulding, Boston, Massachusetts; Spaulding Rehabilitation Hospital, Harvard Medical School, Boston, Massachusetts; Boston University School of Public Health, Rehabilitation Outcomes Center at Spaulding, Boston, Massachusetts; Department of Health Law, Policy and Management, Boston University School of Public Health, Boston, Massachusetts; Shriners Children's-Boston, Massachusetts General Hospital, Harvard Medical School, Boston, Massachusetts; Boston University School of Public Health, Boston, Massachusetts; Phoenix Society for Burn Survivors, Grand Rapids, Michigan; Massachusetts's General Hospital, Boston, Massachusetts; Spaulding Rehabilitation Hospital - Boston Harvard Burn Injury Model System, Boston, Massachusetts; Spaulding Rehabilitation Hospital/Harvard Medical School/Rehabilitation Outcomes Center at Spaulding, Boston, Massachusetts; Spaulding Rehabilitation Hospital, Harvard Medical School, Boston, Massachusetts; Boston University School of Public Health, Rehabilitation Outcomes Center at Spaulding, Boston, Massachusetts; Department of Health Law, Policy and Management, Boston University School of Public Health, Boston, Massachusetts; Shriners Children's-Boston, Massachusetts General Hospital, Harvard Medical School, Boston, Massachusetts; Boston University School of Public Health, Boston, Massachusetts; Phoenix Society for Burn Survivors, Grand Rapids, Michigan; Massachusetts's General Hospital, Boston, Massachusetts; Spaulding Rehabilitation Hospital - Boston Harvard Burn Injury Model System, Boston, Massachusetts

## Abstract

**Introduction:**

Social rehabilitation is a critical step in the recovery process post burn injury, yet there is limited published data on the topic. The LIBRE Profile was developed as a burn specific instrument to examine social participation outcomes across six domains. The objective of this study is to use the LIBRE Profile to measure social participation and explore the trajectories of recovery in each domain.

**Methods:**

Data from the LIBRE Journey Study was analyzed, utilizing both the LIBRE Profile Computer Adaptive Test and fixed short forms. Demographic and clinical characteristics included age, gender, race, ethnicity, education, marital status, burn size and time since injury. LIBRE Profile scores collected at five time-points from baseline to two years, approximately 6 months apart, calculated score trajectories for each of the LIBRE Profile domains (Family and Friends (FF), Social Activities (SA), Romantic Relationships (RR), Sexual Activity (SX), Social Interactions (SI), and Work and Employment (WE)). Linear mixed models with the log of time since burn injury were applied to each domain, adjusting for demographic and clinical variables. Models were fit using the score trajectories with 95% confidence intervals to demonstrate change over time and significant impact of the covariates.

**Results:**

Analyses included 454 participants with a mean age of 45.4 (SD 15.4) and mean burn size of 38.7% (SD 24.6). Time since burn injury ranged from 13 days to 71 years. All participants were included in analyses of the FF, SA, and SI, with 265, 292, and 288 participants included in the WE, RR, and SX domains based on screening questions for relevance, respectively. Multiple predictors were found to significantly effect LIBRE domain scores (Table 1), with all domain scores increasing with time since burn injury (p < 0.0001) and decreasing with an increase in burn size (p < 0.04), except for FF and RR.

**Conclusions:**

Analyses showed significant improvement of social recovery post burn injury for both the short- and long-term in four of the six domains. Analysis of LIBRE Journey data demonstrates the social participation trajectories with findings that can establish future applications in clinical practice.

**Applicability of Research to Practice:**

The LIBRE Profile is a tool used to monitor social participation after burn injury. Understanding the trajectories of social recovery after injury will inform and optimize both clinical care and resource allocation by providing the ability to benchmark an individual’s recovery with one’s peers.

